# Serum Asprosin Concentrations in Children with Prader–Willi Syndrome: Correlations with Metabolic Parameters

**DOI:** 10.3390/jcm11082268

**Published:** 2022-04-18

**Authors:** Maha Alsaif, Catherine J. Field, Eloisa Colin-Ramirez, Carla M. Prado, Andrea M. Haqq

**Affiliations:** 1 Department of Agricultural, Food and Nutritional Science, University of Alberta, Edmonton, AB T6G 2R7, Canada; alsaif@ualberta.ca (M.A.); cjfield@ualberta.ca (C.J.F.); carla.prado@ualberta.ca (C.M.P.); 2 Department of Pediatrics, University of Alberta, Edmonton, AB T6G 2R7, Canada; ecolin@anahuac.mx; 3 School of Sport Sciences, Universidad Anahuac Mexico, Huixquilucan 52786, Mexico

**Keywords:** syndromic obesity, insulin resistance, glucose metabolism

## Abstract

Children with Prader–Willi syndrome (PWS) are characterized by severe obesity. Asprosin is a newly discovered protein hormone produced by the white adipose tissue and is correlated with insulin resistance. The aim of our study was to describe the concentrations of serum asprosin in children with PWS compared to those with overweight/obesity and normal weight, and to explore the postprandial change in asprosin concentrations in participants with PWS and BMI-z matched controls. We enrolled 52 children, 23 with PWS, 8 with overweight/obesity, and 21 with normal weight. Fasting levels of asprosin, glucose, and insulin were collected in all children, and postprandial asprosin and fasting levels of acyl ghrelin (AG) and leptin were also determined in a subsample of participants. There were no significant differences among groups in fasting levels of asprosin, glucose, insulin, and HOMA-IR. Fasting serum asprosin and 1-h post-meal serum asprosin did not differ in children with PWS nor in BMI-z matched controls. Fasting asprosin showed an adjusted positive correlation with glucose in children with obesity (r = 0.93, *p* = 0.007) but not in children with PWS nor children with normal weight. Circulating asprosin might be a predictor of early alterations in glucose metabolism in children with obesity. More research is needed to further explain the association between asprosin, food intake, metabolism, and obesity in PWS.

## 1. Introduction

Prader–Willi syndrome (PWS) is a complex condition that impacts significantly the quality of life of both the affected child and the family [1]. It is a unique clinical model of disordered satiety and paradoxical fasting and postprandial hyperghrelinemia [2]. This condition is characterized by severe obesity in childhood [3], which has been ascribed to several factors, including high circulating concentrations of ghrelin, an orexigenic hormone. Impaired hypothalamic development and function is a contributor to many of the phenotypic characteristics of Prader–Willi syndrome, including excessive weight gain preceding hyperphagia, early severe obesity hormonal deficiencies, behavioral problems, and autonomic nervous system dysfunction [4].

Ghrelin, of which circulating levels are suppressed by food intake, has relevant physiological effects such as stimulation of appetite via central regulating mechanisms in the hypothalamus, stimulation of growth hormone secretion, regulation of energy homeostasis and brown fat thermogenesis and stimulation of gastric emptying. Several studies reported increased ghrelin levels in subjects with PWS compared with BMI-, age-, and sex-matched control children. Ghrelin concentrations also remained elevated even after eating in subjects with PWS compared to non-PWS controls with obesity [5]. Other peptides produced by adipose tissue play an important role in the regulation of both central and peripheral energy metabolism. Leptin, a hormone that has a role in the regulation of appetite and fat storage, seems not to be dysregulated in PWS; no differences in leptin levels between subjects with or without PWS and obesity have been reported [5,6]. In contrast, adiponectin, a hormone involved in regulating obesity, has been found to be lower in subjects with PWS compared with lean controls, but higher compared with subjects with obesity. This hormone has been associated with increased insulin sensitivity in PWS [5,6]. Serum resistin and resistin mRNA expression in adipose tissue was significantly higher in PWS patients, compared with both healthy lean controls and obese patients; however, no significant association has been found between resistin and insulin resistance [5].

Asprosin is a newly discovered protein hormone produced by the white adipose tissue [7]. It stimulates hepatic glucose production via the G protein-coupled receptor (GPCR)-activated cAMP signaling pathway olfactory receptor family 4 subfamily M member 1 (OR4M1) and is correlated with insulin resistance [7]. Additionally, circulating asprosin concentrations increased with fasting in mice, rats, and humans, and decreased with food intake [8]. This suggested that asprosin may act as a circulating orexigenic signal that stimulates appetite and food intake similar to ghrelin by interacting with the neuropeptide tyrosine (NPY)/agouti-related peptide (AgRP) and pro-opiomelanocortin (POMC) pathways [8]. Ghrelin and asprosin seem to activate a partially overlapping subset of AgRP neurons within the arcuate nucleus of the hypothalamus, and asprosin deficiency induces an impaired response by these neurons to ghrelin-mediated activation. However, the role of asprosin and its interplay with other hormones in the energy homeostasis is still unclear and requires further investigation [9].

Fasting asprosin has been measured in adults with metabolic disorders [10]. Wang et al., reported significantly higher concentrations of fasting asprosin in adults with obesity compared to participants without obesity [11]. In children with obesity, fasting asprosin levels have been found to be either increased or decreased compared to those with normal weight, suggesting a complex role of asprosin in pediatric obesity [12]. Asprosin concentrations in children with PWS have not been described. Therefore, the aim of our study was to describe the concentrations of serum asprosin in children with PWS compared to those with overweight/obesity and normal weight, and its correlation with metabolic markers. We also aimed to explore the postprandial change in asprosin concentrations in a subsample of participants with PWS and body mass index (BMI)-z matched controls.

## 2. Materials and Methods

### 2.1. Population

We analyzed data and blood samples from 52 children (23 children with PWS and 29 children with obesity and normal weight), who were enrolled in previous studies. Children with PWS were recruited from the Pediatric Endocrinology Clinic and Stollery Children’s Hospital (Edmonton, AB, Canada). Children with normal weight were siblings or friends of the participants with PWS, while children with overweight/obesity were recruited from the Pediatric Centre for Weight and Health, Edmonton General Continuing Care Centre, Child Health Clinic, Misericordia Hospital (Edmonton, AB, Canada). We excluded individuals with diabetes mellitus, chronic inflammatory bowel disease, chronic severe liver or kidney disease, or neurologic disorders, and those individuals who had been taking an investigational drug in the past year. The study was approved by the University of Alberta’s Health Research Ethics Board (Pro00010454, Pro00009903, Pro00011653, Pro00066276). Written informed consent and assent to participate in the study were obtained from all participants and parents. This study complied with the Declaration of Helsinki regarding ethical conduct of research involving human subjects. A subsample of 10 children with PWS and 7 BMI-z matched controls, who were included in the initial groups and for whom postprandial blood samples were available, was assembled in order to explore postprandial changes in asprosin levels and additional associations of asprosin with metabolic parameters.

### 2.2. Procedures

In a cross-sectional study design, and after confirming eligibility, participants and their parents received instructions for the day of the study visit, including an 8 h fast. On the day of the visit, participants arrived at the study site, where study personnel confirmed with them the time at which the children had their last meal the previous day in order to assure the 8 h fast.

#### 2.2.1. Anthropometry and Body Composition

Weight was measured to the nearest 0.1 kg using a calibrated scale (Pelstar, Bridgeview, IL, USA); height was measured to the nearest 0.1 cm using a Digi-kit stadiometer (Measurement Concepts and Quick Medical, North Bend, WA, USA), and waist circumference (WC) was measured in centimeters (cm) at the top of the iliac crest using standardized techniques. Body mass index-z score was calculated using Epi Info 2000 (CDC, Atlanta, GA, USA; http://www.cdc.gov/epiinfo/, accessed on 14 April 2022). Children were classified as having overweight (BMI-z ≥ +1 standard deviation (SD)), obesity (BMI-z ≥ +2 SD), or normal weight (BMI-z < +1 and >−2 SD). Percentage of total body fat was determined by air displacement plethysmography (BOD POD^®^, Life Measurement Inc., Concord, CA, USA) in the subsample of participants.

#### 2.2.2. Hormone and Adipocytokine Assays

Fasting blood samples to determine levels of asprosin, glucose, and insulin were collected in all children. Fasting levels of acyl ghrelin (AG) and leptin were also determined in the subsample of participants.

In order to assess postprandial levels of asprosin, blood samples were also collected 60 min after consuming a 350 kcal standard meal (SM) breakfast which represents a typical Canadian diet. Fasting and 1 h post-meal serum concentrations of asprosin were measured using an enzyme-linked immunosorbent assay kit, with intra-assay coefficients of variation (CVs) < 10% and inter-assay CVs < 12% (Catalogue No. abx257694; Abbexa, Cambridge, UK). Plasma samples were assayed using a novel sensitive and specific two-site assay for AG in the lab of Dr. Gaylinn [13]. Glucose was measured at Alberta Health Services clinical laboratories, and leptin and insulin levels were determined using RIAs (Millipore). Homeostatic model assessment insulin resistance (HOMA-IR) was calculated as fasting glucose (mg/dL) × fasting insulin (μIU/mL) ÷ 405 [14].

Eighteen participants were taking a stable dose (0.02 mg/kg/d) of recombinant human growth hormone (rhGH) treatment for at least 12 months prior to study enrollment (no changes in GH treatment were made during the course of the study); three had been previously treated with rhGH, but were not taking rhGH at the time of the study; and two had never been treated with GH.

### 2.3. Statistical Analysis

Data are presented as median and interquartile range. Between-group comparisons (PWS, overweight/obesity, and normal weight) for continuous variables were examined using the Kruskal–Wallis H test. Post hoc analyses were performed using the Mann–Whitney U test.

In the subsample of children, between-group comparisons were examined using the Mann–Whitney U test. The Wilcoxon signed-rank test was used for within-group comparison of fasting asprosin and 1 h post-meal asprosin.

Partial correlation analyses were performed to evaluate the relationships between fasting and 1 h postprandial (in the case of the subsample) asprosin with glucose, insulin, HOMA-IR, and WC, adjusting for age and sex, in each group. All statistical analyses were performed using SPSS version 24 for Windows (SPSS Inc., Chicago, IL, USA) and GraphPad Prism 7 software (GraphPad Software Inc., San Diego, CA, USA). A value of *p* < 0.05 was considered statistically significant.

## 3. Results

Characteristics of study participants are shown in Table 1. There were no significant differences among groups in sex distribution, age, and fasting levels of asprosin, glucose, insulin, and HOMA-IR. Median BMI-z was lower in children with PWS compared to children with overweight/obesity (*p* = 0.008), and lower in children with normal weight compared to children with PWS and overweight/obesity (*p* = 0.007 and *p* < 0.001, respectively). Waist circumference was lower in children with normal weight when compared to children with overweight/obesity (*p* = 0.008).

The characteristics of the subsample of participants are presented in Table 2. These data were already reported in a previous study conducted by our research group [15], except for asprosin levels, which did not show significant differences between groups. 

Within-group comparisons of fasting serum asprosin and 1 h post-meal serum asprosin did not differ in children with PWS (3.2 (2.7, 4.5) vs. 3.1 (2.3, 5.0) pg/mL, *p* = 0.58) nor in BMI-z matched controls (4.4 (3.2, 5.4) vs. 3.9 (3.4, 5.8) pg/mL, *p* = 0.87). The median percentage of change in serum asprosin was −3.97% (−11.8, 18.6) in children with PWS and 0.74% (−11.5, 7.4) in BMI-z matched controls (*p* = 1.0).

After adjusting for age and sex, fasting asprosin showed a positive correlation with glucose in children with obesity but not in children with PWS nor children with normal weight (Table 3).

In the subsample analyses, fasting and postprandial asprosin were not significantly correlated with any of the metabolic markers in both children with PWS and BMI-z matched children after adjusting by sex and age (Table 4).

## 4. Discussion

This is the first study to compare fasting asprosin in children with PWS, overweight/obesity, and normal weight. Results of this study showed no differences in the asprosin fasting levels among these study groups. Two studies have reported higher levels of asprosin in children with obesity compared to children with normal weight [12], while Long et al. reported that fasting asprosin levels were lower in children with obesity than in those with normal weight [16]. The lack of association between asprosin and weight status in our study may be related to the limited sample size.

We found no significant differences among the main groups in sex distribution, age, and fasting levels of asprosin, glucose, insulin, and HOMA-IR; however, this may be related, in part, to the lack of power due to the smaller sample size. Long et al. [16] reported that asprosin levels were lower in boys than in girls among children with obesity; thus, we cannot deny the potential effect of sex on asprosin levels and given the effect of age/puberty on insulin resistance, all correlation analyses were adjusted by age and sex.

After adjusting for age and sex, asprosin was positively correlated with glucose in children with obesity, but not in children with PWS or normal weight. This finding is in agreement with Zhang et al., who found a positive correlation between serum asprosin and glucose in adults with T2D [17]. Additionally, in adults with insulin resistance, asprosin concentrations were higher and positively correlated with insulin resistance [18]. In children, Wang et al. observed that those with insulin resistance had higher asprosin levels than children without this condition [12]. It has been suggested that asprosin may be a biomarker contributing to the early diagnosis of diabetes [10].

It has been well documented that children with PWS are more insulin sensitive compared to BMI-matched controls or children with obesity, and it has been associated with higher levels of adiponectin in PWS [6,19]. This may explain why we did not observe a relationship between asprosin and HOMA-IR in children with PWS. In this study, adiponectin levels were not tested. Of note, leptin levels were similar between children with PWS and BMI-z matched controls, whereas AG levels were higher in PWS, which is in line with the metabolic profile described in this population [5]. The interplay between asprosin and key orexigenic and anorexigenic hormones involved in the regulation of obesity in PWS needs to be further investigated.

Importantly, treatment with rhGH, commonly used in PWS to treat GH deficiency, may also alter glucose metabolism [5,20,21]. In this study, most of the children with PWS were taking rhGH; nonetheless, glucose metabolism parameters in these children were lower than in BMI-z matched controls, suggesting that rhGH did not induce impaired glucose tolerance in these children.

A previous study showed that asprosin increased with fasting and decreased after feeding, and that higher asprosin concentrations stimulated appetite in mice models with obesity [22]. In the current study, we did not observe any significant difference between fasting and postprandial asprosin in children with PWS and BMI-z matched controls. Postprandial asprosin was measured one hour after a standard meal was consumed, and the nadir in asprosin may have occurred outside the window of our measurement.

Major strengths of our study include a relatively large sample size for a rare genetic disease with a prevalence of 1 in 15,000 to 30,000 live births [23]. This is the first study to measure asprosin in children with PWS, and postprandial concentration of asprosin. Limitations of our study include the lack of body composition and levels of AG and leptin assessments in the entire cohort, smaller sample size, sex imbalance, lack of pubertal stage data which may impact insulin resistance, and the cross-sectional design.

Much remains to be learned about this hormone, specifically its duration of action. The adjusted association between fasting asprosin and glucose levels in children with obesity suggests that circulating asprosin might be a predictor of early alterations in glucose metabolism, as also supported by other studies in children with obesity [24]. More research is needed to further explain the association between asprosin, food intake, metabolism, and obesity in PWS.

## Figures and Tables

**Table 1:** Characteristics of children with Prader–Willi syndrome (PWS), children with obesity, and children with healthy weight.

Characteristic	PWS (*n* = 23)	Overweight/Obesity (*n* = 8)	Normal Weight (*n* = 21)
Age, y	8.4 (6.2, 12.5)	12.9 (11.1, 15.0)	12.8 (9.0, 14.0)
Males/females	8/15	6/2	13/8
BMI z-score	1.02 (0.3, 1.3) ^a^	1.7 (1.34, 2.1) ^b^	−0.1 (−0.5, 0.4) ^c^
WC, cm	67.7 (55.7, 82.0) ^a,b^	81.43 (76.3, 94.1) ^a^	63.8 (59.6, 70.6) ^b^
Asprosin, pg/mL	3.5 (2.9, 4.5)	4.18 (1.5, 4.5)	3.71 (2.9, 4.4)
Glucose, mmol/L	4.0 (3.7, 4.3)	4.7 (3.2, 4.8)	3.94 (3.6, 4.2)
Insulin, pg/mL	471.5 (95.0, 1754.0)	1738.0 (259.6, 5750.3)	915.5 (289.1, 14)
HOMA-IR	2.0 (0.5, 7.1)	3.5 (2.1, 19.2)	3.7 (1.3, 5.9)

Data reported as medians (interquartile range). Kruskal–Wallis H test for between-groups comparisons, and Mann–Whitney U test for multiple comparison. Groups with different letter superscripts are significantly different (*p* ≤ 0.05) from each other. PWS: Prader–Willi syndrome; y: year; BMI: body mass index; WC: waist circumference; HOMA-IR: homeostatic model assessment insulin resistance.

**Table 2:** Baseline characteristics of children with Prader–Willi syndrome (PWS) and BMI-z matched controls.

Characteristic	PWS (*n* = 10)		BMI-z Matched Controls (*n* = 7)		*p*-Value
Age, y	6.6 (5.4, 10.1)		12.5 (10.2, 13.7)		0.05
Males/females	1/9		6/1		0.002
BMI z-score	1.0 (0.41, 1.2)		0.95 (0.8, 1.1)		0.81
WC, cm	60.0 (54.04, 69.5)		77.45 (68.4, 83)		0.07
Asprosin, pg/mL	3.2 (2.9, 4.3)		4.3 (3.7, 4.9)		0.13
1 hour asprosin, pg/mL	3.1 (2.4, 4.5)		4.9 (3.5, 4.9)		0.16
Glucose, mmol/L	4.35 (4.2, 5.1)		4.7 (3.9, 4.6)		0.04
Insulin, pg/mL	91.85 (77.0, 98.5)		201.9 (110.0, 210.8)		0.13
HOMA-IR	0.43 (0.37, 0.58)		0.9 (0.8, 0.9)		0.05
Acyl ghrelin, pg/mL	189.8 (110.2, 308.9)		88.2 (60.0, 92.6)		0.02
Leptin, ng/mL	7683.7 (43,734.0, 24,142.2)		5058 (2912.4, 5497.8)		0.36
Body fat, %	Male	Females	Males	Female	
	34.8	24.1 (20.7, 34.6)	25.8 (20.8, 28.7)	20.9	0.74

Data reported as medians (interquartile range). Mann–Whitney U test was used for between-groups comparison. PWS: Prader–Willi syndrome; WC: waist circumference; HOMA-IR: homeostatic model assessment insulin resistance.

**Table 3:** Partial correlations of fasting asprosin to other metabolic parameters adjusting for age and sex in children with Prader–Willi syndrome, overweight/obesity, and normal weight.

	PWS (*n* = 23)		Overweight/Obesity (*n* = 8)		Normal Weight (*n* = 21)	
	r	*p*	r	*p*	r	*p*
WC, cm	0.32	0.16	−0.03	0.96	0.22	0.42
Glucose, mmol/L	0.07	0.77	0.93	0.007	0.06	0.79
Insulin, pg/mL	0.02	0.92	−0.78	0.07	−0.20	0.41
HOMA-IR	0.008	0.97	−0.46	0.36	−0.19	0.44

PWS: Prader–Willi syndrome; BMI: body mass index; WC: waist circumference; HOMA-IR: homeostatic model assessment insulin resistance.

**Table 4:** Partial correlation of fasting asprosin to other metabolic parameters adjusting for age and sex in children with Prader–Willi syndrome and BMI-z matched controls.

Fasting Asprosin					1 h Postprandial Asprosin			
	PWS (*n* = 10)		BMI-z Matched Controls (*n* = 7)		PWS (*n* = 10)		BMI-z Matched Controls (*n* = 7)	
	r	*p*	r	*p*	r	*p*	r	*p*
% body fat	−0.20	0.64	0.45	0.45	−0.29	0.49	0.31	0.61
WC, cm	−0.14	0.75	−0.33	0.59	−0.11	0.79	0.19	0.76
Glucose, mmol/L	0.11	0.79	0.34	0.58	0.09	0.83	0.20	0.75
Insulin, pg/mL	−0.37	0.37	0.60	0.28	−0.33	0.42	0.48	0.42
HOMA-IR	−0.13	0.76	0.55	0.34	−0.09	0.84	0.42	0.48
Acyl ghrelin, pg/mL	0.59	0.16	−0.30	0.70	0.49	0.27	−0.29	0.71
Leptin, ng/mL	0.32	0.46	−0.40	0.60	0.36	0.43	−0.44	0.56

PWS: Children with Prader–Willi syndrome; BMI z-s: body mass index z-score; WC: waist circumference; HOMA-IR: homeostatic model assessment insulin resistance.

## Data Availability

The data presented in this study are available on request from the corresponding author. The data are not publicly available due to confidentiality issues.
